# Basket-Type Catheters: Diagnostic Pitfalls Caused by Deformation and Limited Coverage

**DOI:** 10.1155/2016/5340574

**Published:** 2016-12-13

**Authors:** Tobias Oesterlein, Daniel Frisch, Axel Loewe, Gunnar Seemann, Claus Schmitt, Olaf Dössel, Armin Luik

**Affiliations:** ^1^Institute of Biomedical Engineering, Karlsruhe Institute of Technology (KIT), 76131 Karlsruhe, Germany; ^2^Institute for Experimental Cardiovascular Medicine, University Heart Center Freiburg · Bad Krozingen, Medical Center, University of Freiburg, 79106 Freiburg, Germany; ^3^Faculty of Medicine, University of Freiburg, 79106 Freiburg, Germany; ^4^Städtisches Klinikum Karlsruhe, 76133 Karlsruhe, Germany

## Abstract

Whole-chamber mapping using a 64-pole basket catheter (BC) has become a featured approach for the analysis of excitation patterns during atrial fibrillation. A flexible catheter design avoids perforation but may lead to spline bunching and influence coverage. We aim to quantify the catheter deformation and endocardial coverage in clinical situations and study the effect of catheter size and electrode arrangement using an in silico basket model. Atrial coverage and spline separation were evaluated quantitatively in an ensemble of clinical measurements. A computational model of the BC was implemented including an algorithm to adapt its shape to the atrial anatomy. Two clinically relevant mapping positions in each atrium were assessed in both clinical and simulated data. The simulation environment allowed varying both BC size and electrode arrangement. Results showed that interspline distances of more than 20 mm are common, leading to a coverage of less than 50% of the left atrial (LA) surface. In an ideal in silico scenario with variable catheter designs, a maximum coverage of 65% could be reached. As spline bunching and insufficient coverage can hardly be avoided, this has to be taken into account for interpretation of excitation patterns and development of new panoramic mapping techniques.

## 1. Introduction

Panoramic mapping using basket catheters (BCs) offers the unique possibility to simultaneously record the electrical activity of multiple points of the whole atrium. This is an important feature in the analysis of atrial fibrillation, in which the complex spatiotemporal excitation pattern complicates sequential mapping. The Constellation™ basket (Boston Scientific, MA, USA) is a catheter aiming at this objective using 64 electrodes on eight splines. Initial work indicated the potential of this mapping technique in animal studies [1], human atrial flutter [2–4], and atrial fibrillation [5–7]. Besides these panoramic approaches, also local high-density mapping of the pulmonary veins was performed [8, 9]. In order to aid the diagnostic interpretation of this huge amount of simultaneous mapping data, a seminal system for computer-assisted animation was developed [10]. Advances in diagnostically useful algorithms were limited, however, and the BC was not available on the European market in the early 2000s. Recently, the CONFIRM trial demonstrated that the ablation of rotors, recorded with the Constellation catheter, can improve ablation success rate of atrial fibrillation up to 82% [11]. Although the Constellation BC was applied in various research projects addressing onset and mechanisms behind atrial tachycardias, some important aspects were seldom addressed rigorously. First, there are no electrodes located at the proximal end, causing a potential lack of coverage. This was considered a limitation to BC usage [3] and changed in the more recent design of the FIRMap™ catheter (Abbott Electrophysiology, CA, USA). Second, to ensure safety during the procedure, the catheter has a soft and flexible design. Although frequently observed in clinical applications, the resulting spline bunching has not been subject of detailed analysis up to a recent study, in which interspline distances of >8 cm were reported in the equatorial plane [12]. Both coverage and interspline spacing are important aspects when BC mapping data are analyzed. This motivates three goals for this study: (I) First, we want to quantitatively evaluate the effect of catheter deformation in clinical situations, addressing both interspline distance and region specific coverage of the atrial surface. (II) Using an in silico approach, we study the impact of catheter size and electrode configuration on these measures. (III) Finally, we test the hypothesis that neglecting the deformation may lead to misinterpretations of spatiotemporal dynamics of atrial excitation patterns.

## 2. Methods

### 2.1. Mapping Procedure

Nine consecutive patients who underwent basket mapping during the routine ablation procedure for atrial flutter or atrial fibrillation at Städtisches Klinikum Karlsruhe were retrospectively analyzed. All patients provided written informed consent. The EnSite Velocity™ electroanatomical mapping system (St. Jude Medical, MN, USA) was used for data acquisition and ablation guidance. A coronary sinus catheter served as a stable positional reference during the procedure, and respiratory compensation was used to minimize the effect of breathing on catheter tracking. Heparin was administered during the procedure to maintain an activated clotting time of >300 s. Both conventional circular catheters (Lasso™ Biosense Webster, CA, USA; Optima™ and AFocus II™ St. Jude Medical, MN, USA) and basket-type catheters (Constellation Boston Scientific, MA, USA) were used to acquire detailed anatomical shells and electrophysiological information. Previous to the ablation procedure, the routine echocardiography revealed no relevant abnormalities of the right and left atrial anatomy. The mean left atrial diameter of all patients was slightly increased, measured in the parasternal long axis in end systole (for patient details see Supplementary Table T1 in Supplementary Material available online at http://dx.doi.org/10.1155/2016/5340574). The size of the baskets was chosen based on atrial diameter and operator experience. Mapping of the left atrium (LA) was performed in all procedures while the right atrium (RA) was mapped only if deemed necessary. During the electrophysiological (EP) study, the basket was deployed in several locations (including repositioning and rotation) to optimize endocardial contact and signal quality.

#### 2.1.1. Basket Positions

Two positions of the BC were chosen for analysis in each atrium (compare supplemental Figure S1). In our clinical experience, the BC could be placed stably at these positions during data acquisition and exhibited best possible wall contact:

(1) RA anterolateral: catheter pulled back from SVC^*∗*^, orientation bent lateral/anterior, and distal end of basket pointing to RAA^*∗*^/lateral TV^*∗*^ annulus
(2) RA central: catheter pulled back from SVC^*∗*^, orientation kept straight, and distal end of basket pointing towards SVC^*∗*^
(3) LA posterosuperior: distal end pointing towards trigonum LSPV^*∗*^, LIPV^*∗*^, and LAA^*∗*^
(4) LA lateral: distal end pointing towards LAA^*∗*^/MV^*∗*^ annulus

^*∗*^SVC = superior vena cava, RAA = right atrial appendage, LAA = left atrial appendage, TV = tricuspid valve, LSPV = left superior pulmonary vein, LIPV = left inferior pulmonary vein, and MV = mitral valve.

Available data included positions and electrogram information, surface electrocardiogram (ECG), and respiration for at least 10 s, as well as the atrial anatomy of each mapped chamber. They were exported from EnSite Velocity for retrospective analysis.

#### 2.1.2. Preprocessing of Clinical Data

Clinical data were imported for analysis into MATLAB (The MathWorks, MA, USA). We presented details of the analysis previously [13]. Valves and vessels of each atrium were removed to consider just the atrial body and vessel ostia for statistical analysis of coverage. Atrial regions were annotated manually (RA: lateral, posterior, septal, roof, and cavotricuspid isthmus area (CTI); LA: anterior, septal, posterior, roof, right pulmonary vein ostium, and left pulmonary vein ostium). QRS complexes were automatically annotated in the surface ECG [14]. An average position was computed for each electrode based on its locations 100 ms prior to the* R* peaks in the available data segment, in order to minimize the effect of ventricular contraction and respiration on the analysis results.

#### 2.1.3. Modeling Catheter Deformation

A computational model of the BC was implemented in order to study the relationship between its properties (position, diameter, and electrode configuration) and analyzed quantities (interspline distance, coverage). Each opposing pair of splines of the catheter was represented by a parameterized curve **r**(*s*) according to the Frenet-Serret formulas from differential geometry [15]. It was defined by an orthonormal basis spanning *ℝ* ^3^ using tangent **T**, normal **N**, and binormal **B**:

(1) T˙sN˙sB˙sr˙s=0κs00−κs0τs00−τs001000TsNsBsrs.

Splines were discretized by linear segments with curvature *κ*(*s*) and torsion *τ*(*s*) defined in supporting points. The positions of electrodes were given by their spacing along the splines in order to represent a Constellation basket. Dimensions were adapted from data sheets, patents, and specimen.

We developed a computer algorithm which could fit the original basket geometry into the heart model, in order to adapt the catheter shape to a given atrial surface. The algorithm minimized the potential energy of the inner tension to represent a stable catheter position with least deformation. Two nonlinear boundary constraints were applied to account for the geometrical requirements: All supporting points of the splines need to be located inside the cavity and splines need to have a fixed mounting at the distal and the proximal point due to the manufacturing process. We set the initial position and orientation according to the desired mapping scenario with a user interface. Basket shape was then computationally adapted and the positions of all electrodes in space were determined.

An atrial anatomy based on segmented MRI data of a healthy male subject was used for the following in silico studies. The anatomical model was created as described before [16] and converted into a triangulated surface mesh. The atria did not show dilatation (LA distances: anterior to posterior wall 27 mm, septal to lateral wall 51 mm). First, all four clinically relevant mapping positions were evaluated with respect to coverage and spline bunching using an optimally sized basket (48 mm) and two angles of rotation. In a second in silico study, the effect of BC size and electrode configuration was addressed. We modified the basket diameter between 40 mm and 51 mm using increments of 1 mm by adapting the spline length. This covered the range from a nearly undeformed catheter shape to a strong deformation of splines. An alternative design was simulated based on specifications and specimen of a FIRMap catheter (Abbott Electrophysiology, CA, USA). Clamping angle at the shaft and imprinted curvature were adapted accordingly. Importantly, distal and proximal electrodes were located more towards the catheter tip and shaft, respectively, with increased interelectrode spacing along the splines. We will refer to the two electrode arrangements (EA) as Constellation EA and FIRMap EA, respectively (see supplemental Figure S2 for a comparison).

#### 2.1.4. Quantitative Assessment

The deformation of BC and its consequence was measured in clinical and simulated data. To determine the interspline distance, the Euclidean distance between all electrodes of two adjacent splines was computed. For each electrode, the minimum distance to the neighboring spline was retained. The interspline distance was defined as the largest of the minimum distances between electrodes of both splines. The standard deviation of interspline distances for the complete catheter was defined as deformation index. To compute the atrial coverage, the Euclidean distance between each electrode and the closest vertex of the triangulated atrial surface mesh was computed. Electrodes closer than 10 mm were projected onto the atrial surface. An area with a radius of 10 mm around this point was considered as covered surface for the subsequent analysis. The covered surface was computed as percentage of covered endocardium for each anatomical region and for the complete atrium [13]. The quality of wall contact for simulated data was assessed by computing the average distance between electrodes and endocardium.

#### 2.1.5. Simulation of Atrial Fibrillation

An in silico approach was used to demonstrate the effect of spline separation on the outcome of diagnostic algorithms. Based on segmented MRI data of a healthy male subject, a biatrial anatomical model was created as described before [16] (same subject as in Section 2.1.3). The model comprised 1.1 million tissue voxels with isotropic side length of 0.33 mm and was augmented with rule-based fiber architecture [17]. Electrophysiology was represented by a variant of Courtemanche et al. cell model representing chronic AF remodeling [18]. We simulated excitation propagation using the monodomain solver acCELLerate [19] and constant time stepping of 50 *μ*s. The conductance *σ* of the various tissue classes in [16] was uniformly reduced resulting in a longitudinal conduction velocity of 524 mm/s for the free atrial walls. After the system equilibrated in a single cell environment, atrial fibrillation was induced in the organ level simulation. The tissue was preconditioned by an excitation originating from the sinus node. Then, a single premature stimulus of radius 2.5 mm was applied in the right superior pulmonary vein ostium at the end of the refractory phase. This extrasystole initiated AF. The gradient of the transmembrane voltages causes currents that act as the source for the extracellular potential. The extracellular potentials within the atrial cavities could thus be computed in a subsequent forward calculation using a bidomain approach. The simulated atrial fibrillation was virtually mapped using an oversized 60 mm Constellation basket placed in lateral position. The EGMs were extracted at the catheter electrode positions with a sampling frequency of 2 kHz. The phase of EGMs was computed using sinusoidal recomposition and Hilbert transformation [20].

## 3. Results

In all 9 patients (5 male, 4 female, mean age 61 ± 11 years), the BC could be successfully deployed. 22 mapping positions were defined and verified by a second expert. The data were exported and subsequently analyzed. Both 48 mm and 60 mm baskets were used (7 versus 4, including 2 biatrial mappings).

### 3.1. Catheter Deformation in Measured Data

**Figure 1:**
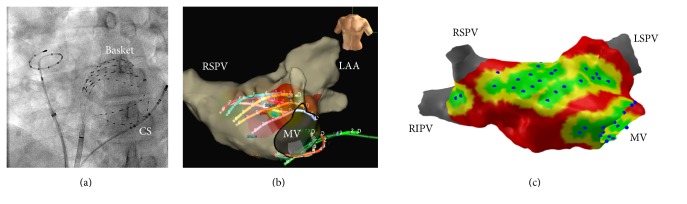
Example for BC deformation. (a) X-ray image of the BC in the LA during the procedure. Note the accumulation of splines at roof and mitral valve annulus. (b) Visualization of the Constellation catheter in the EnSite Velocity system. Eight splines of the basket are visualized in different colors. The CS catheter can be seen in green. (c) The atrial geometry as imported and processed in MATLAB. Blue dots mark the projected surface positions of all electrodes. Areas less than 5 mm away from the electrode surface positions are displayed in green, 5–10 mm in yellow, and >10 mm in red. Areas <10 mm were considered* covered*. All data are from the same patient but do not reflect the same time instance.

We measured the undeformed state of a Constellation basket (60 mm) ex vivo to acquire baseline characteristics. Euclidian interspline distance between the distal electrodes was 11.9 ± 0.6 mm, between the 5th electrodes 20.3 ± 0.9 mm (equatorial plane) and 18.1 ± 0.6 mm for the proximal electrodes, with an interelectrode spacing of 5 mm along the splines. The deformation of a BC due to wall contact can be seen in Figure 1 on both X-ray (a) and the mapping system (b). The resulting coverage map is depicted in (c).

**Figure 2:**
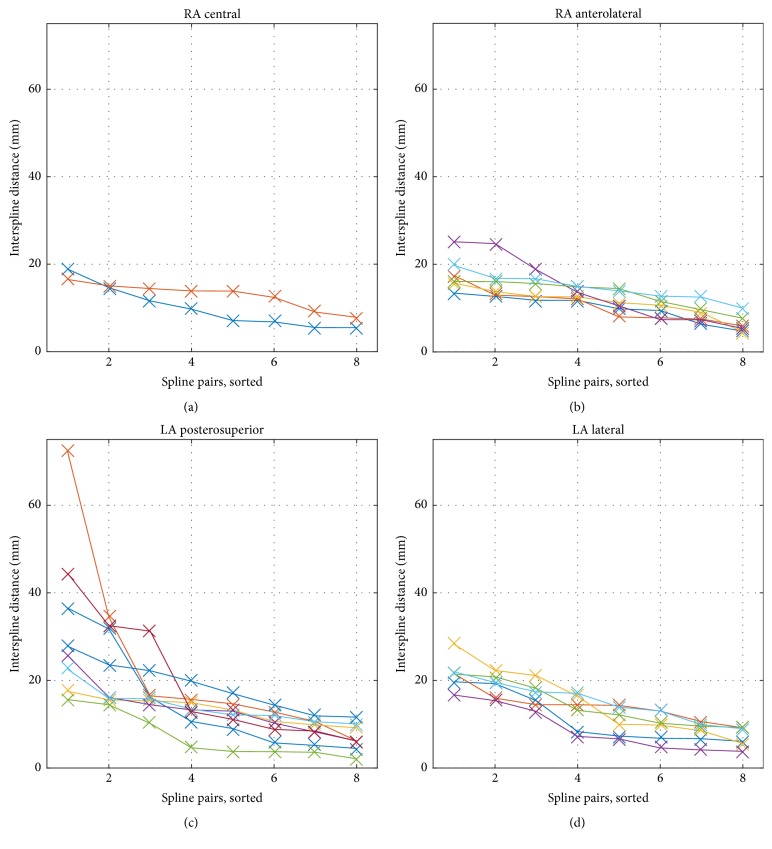
Interspline distances in clinical data. The interspline distance was assessed in all four positions and sorted in decreasing order. Considering RA positions (a, b), the distances between all splines for all except one catheter were below 20 mm, reflecting a rather undeformed state. In both LA positions (c, d), several splines showed distances of more than 20 mm while other splines were close-by with distances below 10 mm.

The interspline distances in clinical data are plotted in Figure 2. The maximum interspline distance was 17.9 ± 3.5 mm and 28.0 ± 15.0 mm for RA and LA positions, respectively. Corresponding mean and std values by catheter type are provided in Table 1. While the distance between splines was similar in RA positions, we observed large variations in the LA, especially in the posterosuperior position.

### 3.2. Atrial Coverage in Clinical Data

**Figure 3:**
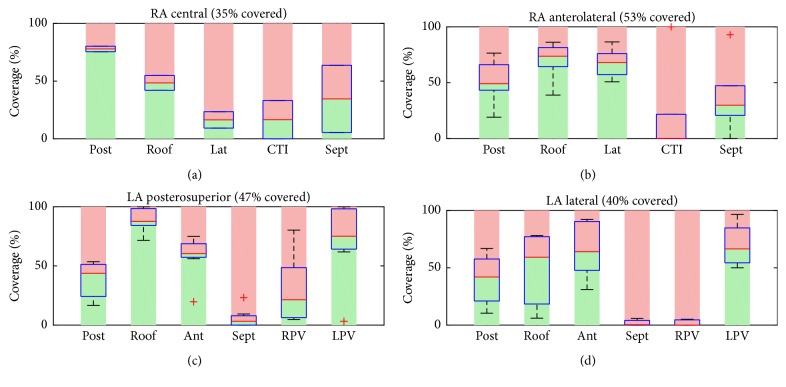
Atrial coverage in clinical data. The median percentage of covered and uncovered tissue is indicated by the green and rose background, respectively. Boxplots indicate the interpatient variability for each atrial region. For the RA, the anterolateral position (b) showed the best coverage with an average of 53%, compared to 35% in the central position (a). In the LA, the posterior-superior position (c) showed a better mean value of 47% which was due to a better coverage of the roof when compared to the lateral orientation (d). Both CTI in the RA and septal areas in the LA were hardly covered.

The coverage achieved in the right and left atrial positions is depicted in Figure 3. RA coverage was higher in the anterolateral position (53%) compared to the central position (35%). Depending on the position, either posterior or lateral areas were covered more comprehensively. We saw little coverage for the inferior part of the RA (CTI < 21%) and septal regions (<37%). For LA positions, the coverage in posterosuperior position was higher than in lateral position (47% versus 40%). Comparable coverage was seen for posterior (39% versus 40%) and anterior (58% versus 65%) wall in both positions. Coverage of the roof decreased in the more lateral position (89% versus 50%). The septum and the antra of the right pulmonary veins were sparsely covered.

### 3.3. Statistics for Virtual Mapping Positions

**Figure 4:**
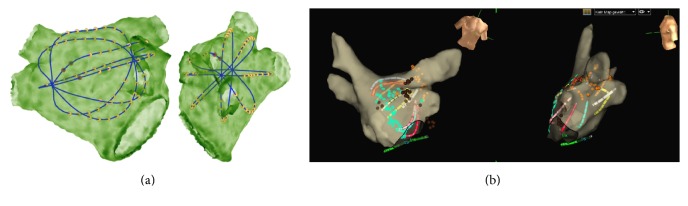
Deformation of a simulated BC. (a) The simulated 48 mm basket was inserted in an MRI-based anatomical model and adapted to the virtual anatomy by the algorithm. (b) Comparison to a clinically observed BC position. Slight deformation and bending of all splines could be observed in both cases.

For demonstration purposes, a computational 48 mm basket with Constellation EA was placed in posterosuperior position in the LA (size 51 mm). The result of shape adaption can be observed in Figure 4(a). A slight deformation from the initial state of equal interspline spacing could be observed. For comparison, a clinical situation is visualized in Figure 4(b).

**Figure 5:**
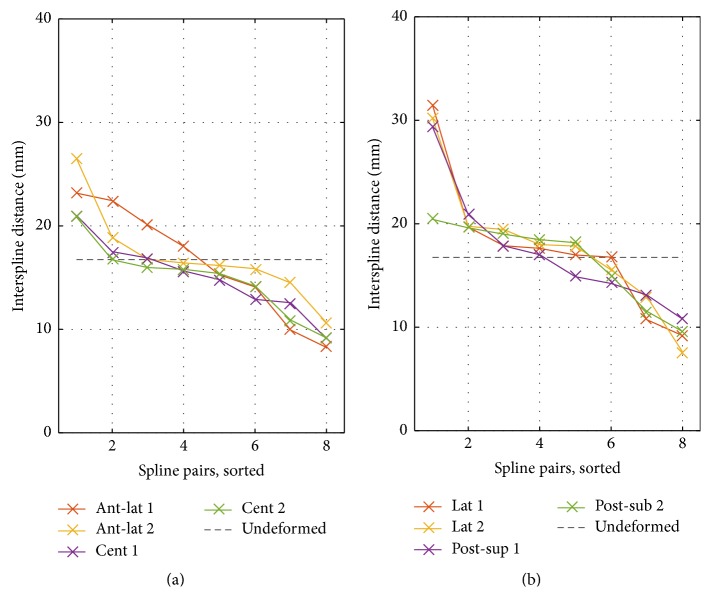
Interspline distances for the simulated 48 mm basket. (a) Basket in RA. (b) Basket in LA. The catheter was positioned in all four mapping positions and rotated by half the interspline angle (1 versus 2). Spline pairs are sorted for descending values. The interspline distance of the undeformed catheter is plotted as reference (dashed line). Results showed a deformation of the catheter due to the wall contact leading to varying interspline distance.

For statistical evaluation, the interspline distance was computed for both positions and two angles of rotation in each atrium (Figure 5). Interspline distances larger and smaller than in the undeformed state (dashed line) can be observed, indicating the presence of spline bunching. Maximum interspline distances of these four positions were higher in the LA than in the RA (28 ± 5.0 mm versus 23 ± 2.6 mm).

### 3.4. Effect of Basket Size and Electrode Arrangement

**Figure 6:**
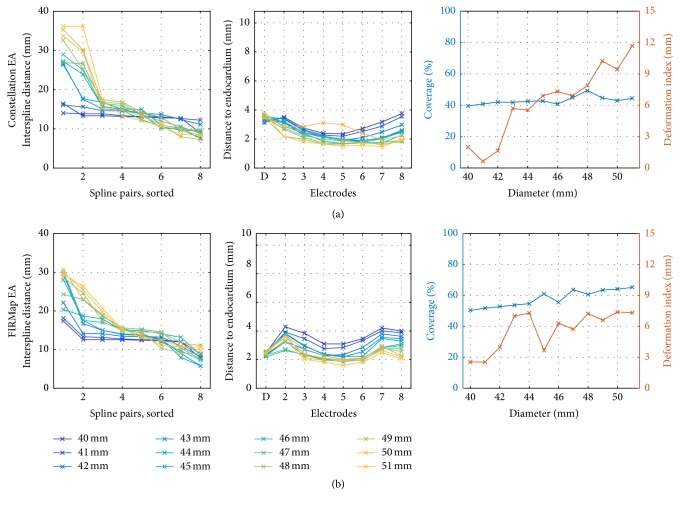
Effect of catheter diameter and electrode arrangement. The virtual catheter size was varied from 40 mm to 51 mm for both the Constellation EA (a) and the FIRMap EA (b). (Left) The interspline distance was similar for all splines for little catheter size, while its variability increased with increasing catheter size. (Middle) An increasing catheter size caused a better contact of more electrodes. (Right) Both resulting coverage and deformation generally increased with catheter size. About 50% of atrial surface could be covered for the Constellation EA in commercially available size 48 mm and 64% for the FIRMap EA size 50 mm.

The influence of BC diameter on interspline distance and atrial coverage was evaluated by changing the spline length of the computational BC (see Section 2.1.3). Concordantly to the LA size of 51 mm, we varied the size of the BC between 40 mm and 51 mm in steps of 1 mm. This study was performed for both Constellation EA and FIRMap EA. For an undersized catheter, the interspline distance was nearly constant, indicating that almost no deformation occurred (Figure 6 left, blue lines). With increasing size, however, the splines tended to move apart, leading to distances of more than twice the initial value (yellow lines). Distances from the electrodes to the endocardium decreased on average with increasing catheter size (Figure 6 middle). We observed an increase of total coverage with size for the Constellation EA (40% to 49%, Figure 6(a) right) and for the FIRMap EA (50% to 65%, Figure 6(b) right). For the FIRMap EA spacing, a smaller distance to the endocardium could be observed for the distal electrodes since they were closer to the catheter tip and thus more close to the lateral wall (Figure 6(b) middle).

**Figure 7:**
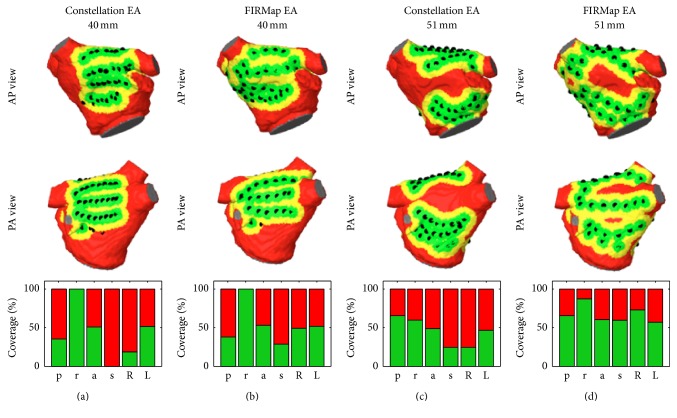
Region specific coverage for under- and oversized baskets. Due to the lack of septal electrodes, the Constellation EA (40%, (a)) showed smaller coverage than the FIRMap EA (50%, (b)). For the 51 mm oversized baskets, parts of the anterior and the posterior wall remained uncovered. Total coverage, however, increased to 44% (Constellation EA, (c)) and 65% (FIRMap EA, (d)). Percentage of region specific coverage is depicted in the lower row (*p: posterior wall, r: roof, a: anterior wall, s: septum, R: right PV ostium, and L: left PV ostium*). Green/yellow = covered and red = uncovered. AP = anterior posterior and PA = posterior anterior.

The smallest and the largest catheters were plotted for visual inspection (Figure 7). The effects of spline separation could be observed when comparing small and large diameters, leading to spline bunching and uncovered regions on both the anterior and the posterior wall. The relative coverage of atrial regions is plotted in the lower row. Results indicate that the septal area was better covered using the FIRMap electrode arrangement. The amount of covered area of the roof decreased when the splines bunched in this area.

### 3.5. Mapping Cardiac Activation Patterns

**Figure 8:**
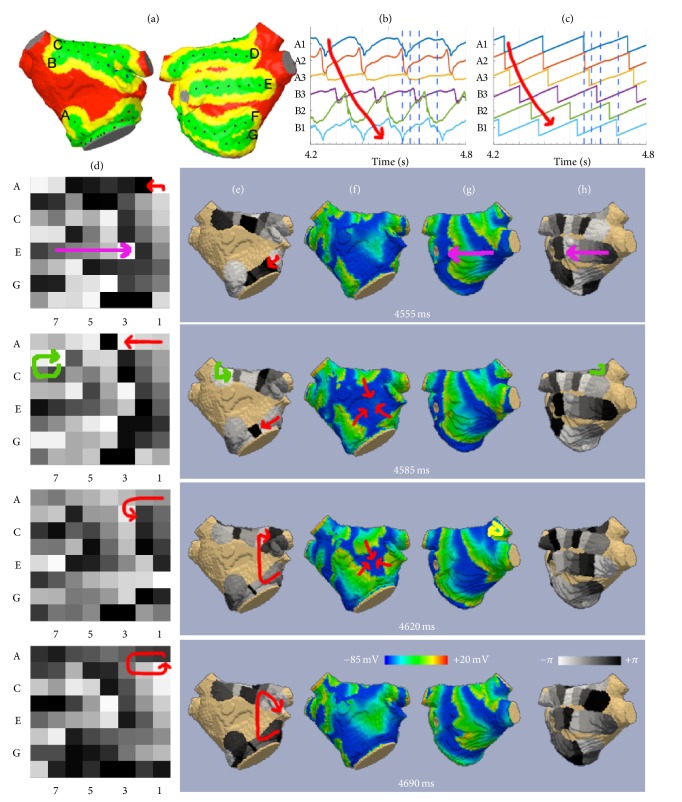
Virtual mapping using a computational BC. The simulated coverage is depicted in panel (a). Unipolar signals from electrodes 1–3 of splines A and B indicate a rotational pattern (b), which is more clearly visible in phase space (c). The complete phase maps were plotted for four time instances, in which a rotational pattern can be observed at distal A/B ((d), red arrow) and proximal B/C ((d), green arrow). Excitation of the posterior wall by broad wave fronts (purple arrow) was well reflected in the phase maps, while one-half of the driving source remained outside the mapped area (yellow arrow). Visualizing the phase map on the atrial shell allowed recognizing a considerable distance between splines A and B (e, h), indicating that an additional mapping of the uncovered region may be reasonable. Indeed, the transmembrane voltage showed a wave collision on the anterior wall (f, g), which may have been misinterpreted as a rotational driver without knowledge about spline separation.

We mapped a sequence of simulated atrial fibrillation virtually using a 60 mm BC model with Constellation EA, focusing on potentially diagnostically relevant effects of spline separation. After the shape was adapted to the anatomy, basket spline A had moved to the anterior MV annulus, spline B to the high anterior wall, and so on. As shown in Figure 8(a), the posterior wall was well covered, while spline bunching caused a lack of coverage on the anterior wall.

EGMs of 6 exemplary electrodes (splines A and B, electrodes 1–3) are shown in Figure 8(b), sorted in circular order (A1, A2, A3, B3, B2, and B1). The corresponding phase indicated a gradient in activation which persisted for several cycles (Figure 8(c)).

The complete phase map of all 64 electrodes is shown in Figure 8(d) for 4 time steps, and the rotational counterclockwise progression of phase can be observed around distal A/B and proximal B/C. A 3D projection of the phase on the atrial shell is plotted in Figure 8(e) (anterior aspect) and Figure 8(h) (posterior aspect). For both 2D and 3D, the value range −*π* to *π* of phase is given in gray-scale. The step from 2D to 3D can be followed best by spline-wise comparison (e.g., spline A located at anterior MV annulus, spline E at central posterior wall). In order to minimize interpolation, the phase is only plotted in the field of view of each respective electrode. Thus septal and central anterior areas remain vacant and a lack of information for the region between splines A and B becomes apparent in the 3D view (see supplementary videos for the animated sequences in 2D and 3D).

For comparison with the simulated ground truth, the transmembrane voltage (TMV) is plotted in Figures 8(f) and 8(g). While blue indicates repolarized myocardium (TMV ≈ −80 mV), the excitation wave can be recognized in yellow (TMV ≈ 0 mV). Detailed inspection of the simulated fibrillation process revealed that it was perpetuated by a Figure-of-8 reentry, which was located at the posterior ostium of the right superior pulmonary vein. While the proximal electrodes on splines B/C did acquire one part of the reentry movement, the other half was not covered by the BC. The posterior wall was dominantly depolarized by broad wave fronts running from septal to lateral, which was well reflected by the panoramic mapping. The anterior wall was activated by three wave fronts coming from the roof, lateral MV annulus, and septal MV annulus and colliding at the central anterior wall. This process was not adequately sampled by the BC due to the spread of splines and could thus been misinterpreted as a rotational driver.

The analysis of the 2D phase map (Figure 8(d)) was based only on EGM information and not including the electrode positions. Thus the increased interspline distance was not considered and the lack of information could not be detected.

## 4. Discussion

### 4.1. Basket Deformation in Clinical Data

In this study, we quantified coverage of the atrial chambers in clinical mapping data. For four selected right and left atrial positions, a maximum coverage of 53% (RA anterolateral) and 47% (LA posterior-superior) was achieved, respectively, as shown in Figure 3. Comparing region specific values for central and anterolateral position in the RA, the latter demonstrated increased lateral and anterior coverage, while coverage of the posterior wall decreased. CTI was hardly covered in both positions. Both septal and RPV ostial regions were hardly covered in LA basket positions. Major differences in region specific coverage between posterosuperior and lateral position were found on the roof, which was less covered for the latter. While only 1 of 6 (17%) RA anterolateral positions did show maximum interspline distances exceeding 20 mm, this increased to 6 of 8 (75%) LA posterosuperior positions (cf. Figure 2).

These phenomena are in agreement with observations by other groups [3, 12, 21, 22]. The lack of coverage in CTI and septal regions was already indicated in early work [3]. In an analysis of 25 patients from the STARLIGHT study, spline bunching was regularly observed in the left atrium with 2 splines being located at the mitral valve annulus and 4-5 splines grouping in the LA roof. Only 1/3 to 2/3 of electrodes were considered usable for LAT mapping, which was not improved by repeated deployment maneuvers [12]. In a study addressing the incidence of phase singularities in BC mapping data, a coverage of 43 ± 16% in the LA and of 60 ± 23% was reported in a cohort of 19 patients [21], being in agreement with our findings. Only 54 ± 15% of LA surface was found located closer than 10 mm to any Constellation basket electrode in a study addressing electrograms characteristics at rotor sites [22].

### 4.2. Lessons Learned from Clinical and Simulated Data

Several findings from the simulation studies are corresponding to clinical observations. First, the bunching of splines as reported [12] could be reproduced as an outcome of shape adaption by modeling. LA positions caused stronger spline separation with an accumulation in roof and mitral valve areas (see Figure 7). This is due to the anatomical rather flat shape of the LA when compared to RA. Increasing catheter diameter resulted in an increased tendency to pronounced spline bunching.

Second, the analysis of coverage with respect to several positions, rotations, and catheter sizes in the LA leads to similar values for both clinical and simulated cases. About 40% to 65% of endocardial surface was covered as shown in Figure 6. For the Constellation EA, the best coverage was 49% (considering the available 48 mm basket). For the FIRMap EA, 64% was achieved using the 50 mm size.

### 4.3. Limitations of BC Mapping

Basket mapping is considered as a full contact mapping of the whole atrial chambers. The catheters are available in diameters of 31 mm, 38 mm, 48 mm, 60 mm, and 75 mm, allowing for individual sizing. One limitation of the Constellation catheter is the lack of septal electrodes, making it hard to cover the inferior part of the right atrium (CTI) and the septal part of the left atrium (cf. Figure 3) [3]. This issue was addressed by the development of an alternative design for the FIRMap catheter, which is available in sizes of 50 mm, 60 mm, and 70 mm. For the clinical setting, an ideal basket might not always be available and the basket is under- or oversized. This may reduce the maximum achievable coverage.

Another limiting factor is the individual anatomy. If the atrial shape is rather flat or the BC is chosen too large, accumulation of splines can be observed in both the roof and the MV area. This was reflected by interspline distances above 20 mm, while other splines were less than 10 mm apart.

### 4.4. Catheter Size, Position, and Electrode Arrangement

The analysis of clinical data indicated that the posterosuperior position in the LA provides better coverage than the lateral position. Achievable stable positions, however, may depend on the individual patients anatomy. The catheter size is usually chosen based on LA size as measured from CT, MRI, or echocardiography. The FIRMap EA was shown to provide more coverage in the septal area. However, total number of electrodes is the same and thus interelectrode distance is increased, causing a decrease in mapping resolution. In general, simulations showed better coverage for FIRMap EA and a trend to better coverage with larger catheter size. An increase of catheter size also leads to uncovered areas on the posterior and the anterior wall. However, using a slightly oversized catheter seems reasonable. The resulting coverage and measurement positions should be taken into account during clinical diagnosis.

### 4.5. Impact on Diagnosis

Acquiring electrograms with intracardiac catheters basically means to observe the cardiac excitation at the electrodes positions. This corresponds to sampling the propagation pattern at defined locations. Compared to atrial flutter, a higher density of electrode locations is required to sample the complex excitation patterns during atrial fibrillation.

Theoretical considerations and computational modeling have shown that a temporal resolution of 1 ms and a spatial resolution of 10 mm can be considered sufficient to capture AF rotors and focal sources [23]. Based on the interspline distances reported in our work and other groups, these criteria are not always fulfilled for all spline pairs of the BC. Notably, any fully deployed BC with a diameter of 50 mm has an interspline distance of about 20 mm.

The identification of phase singularities was recently addressed in a study using high-density epicardial mapping data [24]. A grid of 16 × 16 electrodes with 1.5 mm interelectrode spacing was analyzed by considering different levels of spatial resolution. Results indicated a growing number of detected phase singularities with increasing interelectrode spacing.

These considerations make both coverage and interspline spacing important aspects when BC mapping data are analyzed. The distances between electrodes were already incorporated into dedicated processing algorithms [25]. Simplification on standardized 2D maps without information about the distance between splines and assuming an equal distribution of the electrodes may lead to an overestimation of rotational sources as demonstrated in Figure 8. This may stipulate the remapping of potential rotor sites using other approaches for confirmation. The three-dimensional structure of the atria and distribution of measurements represent important aspects in various processing techniques [13, 25–27].

### 4.6. Clinical Relevance

Panoramic mapping using BCs is the primary source of data for analysis techniques like FIRMap guided procedures. Narayan et al. demonstrated an improved ablation success rate of atrial fibrillation up to 82% [11], fostering intense discussions [28], and reports about case series [29]. These success rates could not be reproduced by all other groups [30, 31].

The presented study emphasizes the need for very carefully monitoring the deployment and recording positions when using the BC. Data which were acquired in an insufficient way cannot be reconstructed during analysis.

The rotors identified during FIRM guided procedures have been used as basis for consecutive studies addressing relation between rotors and complex fractionated atrial electrograms [32], entropy [22], and fibrosis [33]. Indicating the position of rotors in the form of their respective spline and electrode pairs may consequently refer to a rather broad atrial area and complicate the selective remapping of* rotor potentials*. A more detailed multipolar remapping and analysis of the respective areas seems reasonable [34].

### 4.7. Limitations

The clinical data about basket deformation were retrospectively selected from a single center. Thus, a bias due to operator dependency cannot be excluded. Each patient who was mapped using a BC was included. However, the analysis of a larger patient cohort including multiple centers would be beneficial. Data for the central RA position was limited with only two cases. Clinical data was acquired using the Constellation catheter, which is one out of two panoramic baskets currently on the market. Additional clinical data from recordings with the FIRMap catheter would allow to compare the extent of deformation between both types but was not available yet. Only a maximum number of 128 channels could be tracked and recorded by the Velocity system, limiting the technical capabilities in simultaneous biatrial mapping. For statistical analysis, the location of up to three unconnected or compromised electrodes was interpolated based on locations of neighboring electrodes for three patients. Despite advantages in localization and a benchmarked accuracy of 0.7 ± 1.5 mm [35], the impedance-based mapping system Velocity may have a certain error in spatial information. The impact of these potential errors was kept low by comparing relative quantities for coverage and verification of huge deformations by X-ray. The maximum distance between electrode and endocardium was set to 10 mm and the radius of coverage to 10 mm, being conservative values also used by other groups [21, 22]. Consequently, more restrictive settings like a radius of coverage of 5 mm are expected to reduce the resulting coverage.

With respect to the virtual basket, the material properties of the catheter splines were set flexible and the atrial geometry rigid. This is in agreement with the catheter design being highly adaptive to the atrial anatomy. Geometry and electrode positions were adapted for the FIRMap EA configuration, leaving spline flexibility unmodified. The range of catheter sizes from 40 mm to 51 mm was selected to cover a reasonable value range for this anatomy, despite the fact that they are not commercially available. Although the MRI-based atrial geometry used for simulations is suspected to be sufficiently accurate, the application on several anatomical models would increase the statistical reliability of the results.

## 5. Conclusions

First, the left atrial coverage in clinical BC data was shown to be lower than 50% with maximum interspline distances frequently higher than 20 mm. This is a potential pitfall for diagnosis since a spatial resolution of less than 10 mm is considered necessary for the detection of rotors. Care should be taken by the operator to position the basket in an optimal way providing best achievable coverage and minimizing spline deformation.

Second, the simulation results from this study illustrate the tradeoff between catheter deformation and wall contact, which may influence catheter selection. Our results indicate that a slightly oversized basket seems appropriate.

Third, the authors want to raise attention about how combined computational modeling of arrhythmias and catheters may aid in developing advanced algorithms for the analysis of intracardiac mapping data. Benchmarking of new techniques in a simulation study may help to identify limitations and formulate requirements for applicability. Since the amount of acquired data during BC mapping is huge, we do fully agree that simplification is required to visualize the important findings of electrograms analysis. However, we suggest that a kind of trustability measure should be included in the analysis, indicating whenever the sampling density is not adequate for the observed process.

## Supplementary Material

Supplemental Table T1: Patient characteristics of the study cohort.
Supplemental Figure S1: Sketch of all four analyzed basket catheter positions.
Supplemental Figure S2: Photographs of both implemented catheter types: Constellation and FIRMap.
Supplemental Video S1: Video of the phase movie in 2D. Phases were computed for 600 ms of data during simulated atrial fibrillation.
Supplemental Video S2: Video of the phase movie in 3D. The phase values are taken from supplemental video S1 but projected onto the atrial anatomy based on the actual recording positions.

## Figures and Tables

**Table 1:** Statistics for the interspline distance. Mean ± standard deviation of interspline distances depending on mapping position and catheter size. Values from all considered spline pairs are given in mm. Values in parenthesis indicate the number of measurements included.

	Position	48 mm basket	60 mm basket
RA	Central	12.9 ± 3.0 (8)	10.0 ± 4.8 (8)
RA	Anterolateral	11.9 ± 3.6 (40)	14.1 ± 7.9 (8)
LA	Posterosuperior	15.7 ± 7.9 (40)	15.0 ± 15.5 (24)
LA	Lateral	14.7 ± 5.3 (32)	10.0 ± 5.5 (16)
