# Supplementary material for: Basket-Type Catheters: Diagnostic Pitfalls Caused by Deformation and Limited Coverage
Source: Biomed Res Int. 2016 Dec 13;2016:5340574. doi: 10.1155/2016/5340574 (PMC5187596; doi:10.1155/2016/5340574)
Supplement: Supplementary file 1 — Supplemental Table T1: Patient characteristics of the study cohort. Supplemental Figure S1: Sketch of all four analyzed basket catheter positions. Supplemental Figure S2: Photographs of both implemented catheter types: Constellation and FIRMap. Supplemental Video S1: Video of the phase movie in 2D. Phases were computed for 600 ms of data during simulated atrial fibrillation. Supplemental Video S2: Video of the phase movie in 3D. The phase values are taken from supplemental video S1 but projected onto the atrial anatomy based on the actual recording positions. [file 5340574.f1.pdf]

Supplemental Table T1

Patient characteristics	
Age (years)	61 ± 11
Female (n)	4 (44%)
Body mass index	26 ± 6
Paroxysmal AF (n)	2 (22%)
Persistent AF (n)	5 (56%)
Atypical atrial flutter (n)	2…(22%)
Left atrial diameter (mm)	41 ± 3
Left ventricular ejection fraction (%)	64 ± 2
Mild mitral regurgitation (n)	7 (78%)
Coronary artery disease (n)	1 (11%)
Hypertension (n)	6 (67%)
Diabetes mellitus (n)	2 (22%)

Age, body mass index, left atrial diameter and left ventricular ejection fraction are given in mean and standard deviation values. The left atrial diameter was measured by echocardiography in a parasternal long axis view in end systole. All other values are given in total numbers and percentages.
