# Supplementary material for: Basket-Type Catheters: Diagnostic Pitfalls Caused by Deformation and Limited Coverage
Source: Biomed Res Int. 2016 Dec 13;2016:5340574. doi: 10.1155/2016/5340574 (PMC5187596; doi:10.1155/2016/5340574)
Supplement: Supplementary file 2 [file 5340574.f2.pdf]

Supplemental Figure S1

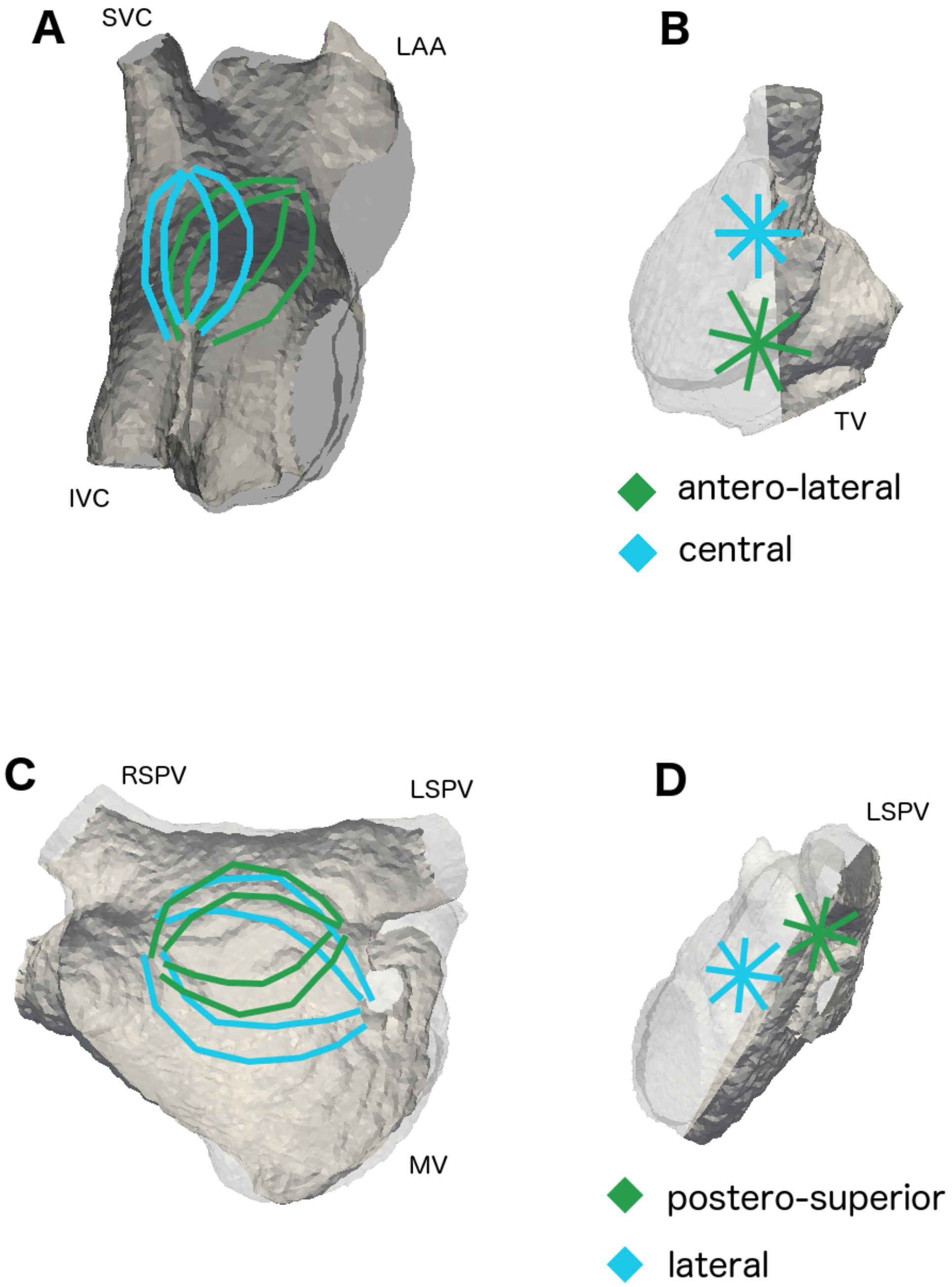

Sketch of basket positions as described in Chapter 2.1.1. Right atrial positions are shown from right lateral (**A**) and cranio-caudal (**B**) perspective. Left atrial positions are shown from anterior (**C**) and left lateral (**D**) perspective. Anatomies on the left were cut open to visualize the catheter positions. The cutting planes are indicated on the right hand side by reduced opacity. *(SVC: Superior Vena Cava; IVC: Inferior Vena Cava; LAA: Left Atrial Appendage; TV: Tricuspid Valve; RSPV: Right Superior Pulmonary Vein; LSPV: Left Superior Pulmonary Vein, MV: Mitral Valve))*
