# Supplementary material for: Basket-Type Catheters: Diagnostic Pitfalls Caused by Deformation and Limited Coverage
Source: Biomed Res Int. 2016 Dec 13;2016:5340574. doi: 10.1155/2016/5340574 (PMC5187596; doi:10.1155/2016/5340574)

Supplemental Figure S2

Two types of basket catheters for panoramic mapping were assessed:

Constellation

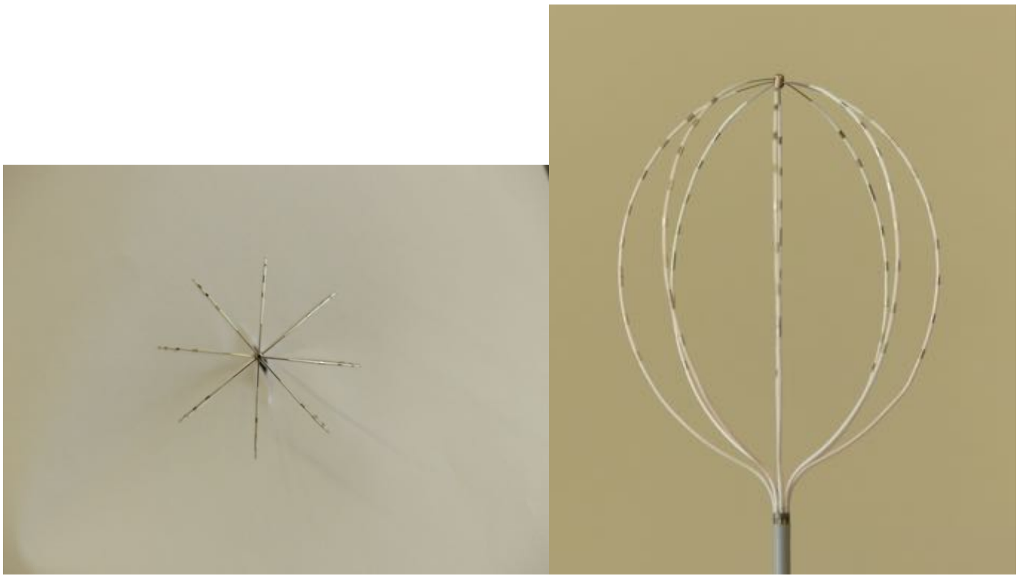

FIRMap

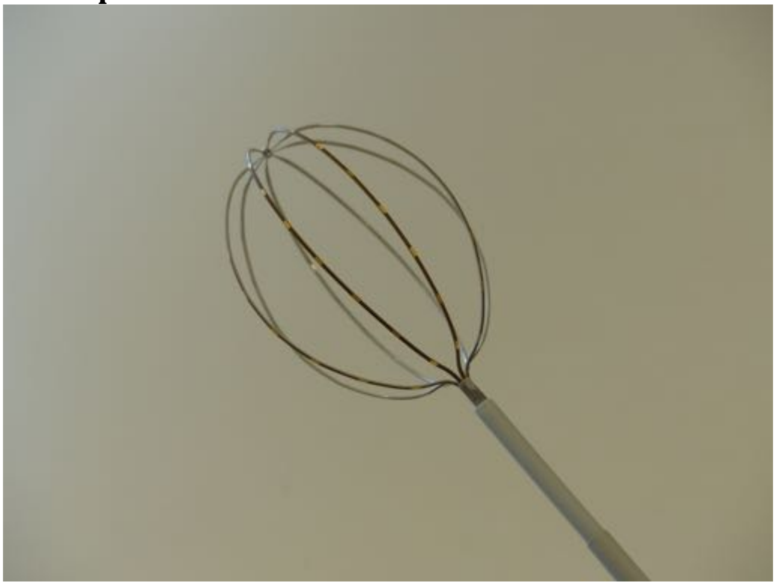

Supplement: Supplementary file 3 [file 5340574.f3.pdf]
